# Home Pulse Pressure Predicts Death and Cardiovascular Events in Peritoneal Dialysis Patients

**DOI:** 10.3390/jcm12123904

**Published:** 2023-06-07

**Authors:** Vincenzo Panuccio, Pasquale Fabio Provenzano, Rocco Tripepi, Maria Carmela Versace, Giovanna Parlongo, Emma Politi, Antonio Vilasi, Sabrina Mezzatesta, Domenico Abelardo, Giovanni Luigi Tripepi, Claudia Torino

**Affiliations:** 1 Nephology, Dialysis and Transplantation Unit—GOM “Bianchi-Melacrino-Morelli”, Via Vallone Petrara SNC, 89124 Reggio Calabria, Italy; enzopanuccio@gmail.com (V.P.); giovy.parl@gmail.com (G.P.); emma.politi@gmail.com (E.P.); 2 National Research Council—Institute of Clinical Physiology, Via Vallone Petrara SNC, 89124 Reggio Calabria, Italy; fprovenzano@ifc.cnr.it (P.F.P.); rtripepi@ifc.cnr.it (R.T.); mcversace@ifc.cnr.it (M.C.V.); avilasi@ifc.cnr.it (A.V.); smezzatesta@ifc.cnr.it (S.M.); dabelardo@ifc.cnr.it (D.A.); gtripepi@ifc.cnr.it (G.L.T.)

**Keywords:** home pulse pressure, peritoneal dialysis, ESKD

## Abstract

Increased arterial hypertension represents a prevalent condition in peritoneal dialysis patients that is often related to volume expansion. Pulse pressure is a robust predictor of mortality in dialysis patients, but its association with mortality is unknown in peritoneal patients. We investigated the relationship between home pulse pressure and survival in 140 PD patients. During a mean follow-up of 35 months, 62 patients died, and 66 experienced the combined event death/CV events. In a crude COX regression analysis, a five-unit increase in HPP was associated with a 17% increase in the hazard ratio of mortality (HR: 1.17, 95% CI 1.08–1.26 *p* < 0.001). This result was confirmed in a multiple Cox model adjusted for age, gender, diabetes, systolic arterial pressure, and dialysis adequacy (HR: 1.31, 95% CI 1.12–1.52, *p* = 0.001). Similar results were obtained considering the combined event death–CV events as an outcome. Home pulse pressure represents, in part, arterial stiffness, and it is strongly related to all-cause mortality in peritoneal patients. In these high cardiovascular risk populations, it is important to maintain optimal blood pressure control, but it is fundamental to consider all the other cardiovascular risk indicators, such as pulse pressure. Home pulse pressure measurement is easy and feasible and can add important information for the identification and management of high-risk patients.

## 1. Introduction

Chronic kidney disease (CKD) is a global health issue. The disease burden has noticeably risen in recent years, with an estimated increase in CKD-related years of life lost equal to 82% [1]. Among the comorbidities affecting these patients, hypertension has a prevalence twice higher than in the general population [2,3]. In addition, it is more frequently resistant to drug treatment [4], and it exhibits a circadian profile with an attenuated or abolished decline during night-time [5,6]. Different from the general population, in which blood pressure (BP) shows a linear, direct relationship with cardiovascular events [7,8,9], this association is more complex in these patients [10,11,12,13]. Data reported in the literature suggests that both low [14,15,16,17,18] and high peri-dialysis BP are associated with adverse outcomes [19,20,21], thus indicating a U-shaped association between BP, fatal and non-fatal CV events, and mortality [5,15,20,22,23,24,25,26,27]. Based on these observations, several research groups started pointing the finger against peri-dialytic blood pressure measurements, highlighting their high variability in the absence of standardized measurements [28], their poor agreement with inter-dialytic measurements [29,30,31], and the lack of association with organ damage, such as left ventricular hypertrophy [32].

Different from peri-dialysis measurements, out-of-dialysis BP is more efficient in diagnosing hypertension [29] and in predicting organ damage [29,32,33] in hemodialysis (HD) patients. Most importantly, it shows a direct and linear association with mortality and cardiovascular outcomes [31,34,35] and a predictive power for death and cardiovascular outcomes, which overcomes that of pre- and post-dialysis measurements [31,34,35,36,37].

Among the out-of-dialysis measurements, 44 h ambulatory blood pressure measurement (ABPM) is the gold standard for the diagnosis of hypertension in dialysis patients; however, its applicability and tolerability are still a matter of debate [38,39]. For this reason, home BP monitoring is considered a more suitable option for the management of hypertension in hemodialysis patients [40,41].

Home BP monitoring represents the election choice for peritoneal dialysis (PD) patients who, due to the home-based treatment, routinely measure BP at home. However, information about the diagnostic accuracy of this measurement in these patients is scarce and in contrast with findings in HD patients. Among the studies published so far, most of them with limited sample size suggest an overestimation of daytime ambulatory BP and a worse performance in diagnosing hypertension compared with standardized BP [42,43]. More recently, a study performed on 81 PD patients showed a similar accuracy for 1-week averaged home BP and standardized clinic SBP in diagnosing hypertension [44]. To date, fewer studies have focused on the association between BP and clinical outcomes in PD patients [45,46,47]. However, as all these studies considered clinical, standardized BP measurements, to our knowledge, no evidence is currently available on the association between home BP and clinical outcomes in PD patients. Increased pulse pressure (PP), a component of blood pressure, is frequently observed in chronic renal failure patients [48,49], and it is associated with decreased arterial-wall compliance [50]. PP is a robust predictor of mortality in dialysis patients [51,52], but the association between home pulse pressure (HPP) and mortality is still unknown in dialysis patients. With this background in mind, we tested this hypothesis in a cohort of PD patients in a single unit in Calabria, Italy.

## 2. Materials and Methods

This study was conducted in accordance with the Declaration of Helsinki, and the protocol was approved by the Ethical Committee of our institution (Sezione Sud—Regione Calabria). Each participant provided written informed consent before enrolment.

### 2.1. Study Population

We included in this analysis all patients who started peritoneal dialysis at the Nephrology, Dialysis and Transplantation Unit of the Grande Ospedale Metropolitano “Bianchi-Melacrino-Morelli”, Italy, between August 1991 and December 2018 (*n* = 140). All the enrolled subjects were of Caucasian descent and came from the same geographic area (Southern Italy). After the baseline visit, patients were followed up for a median time of 33 months (range: 20–58 months). One-hundred and six patients were treated with various anti-hypertensive drugs (33 on mono-therapy with ACE inhibitors, calcium channel blockers, α- and β-blockers, vasodilators, diuretics or other drugs, 31 on double therapy, 26 on triple therapy, 10 on quadruple therapy, and 6 patients on quintuple or sextuple therapy, with various combinations of these drugs). The main demographic, somatometric, clinical, and biochemical characteristics of the study population are detailed in Table 1.

### 2.2. Clinical and Laboratory Measurements

Blood pressure was measured at home once a day, in the morning, before dialysis exchange, with an automatic blood pressure monitor. To perform the measurements, any monitor owned by the patients was allowed, provided it was an automatic upper-arm monitor that met the AHA standards. Detailed instructions on how to measure BP, with recommendations regarding the hydration status or the risk of infections, were provided to the patients during the peritoneal training period. Briefly, patients were recommended to measure blood pressure seated, after 5 min of rest, only once, and to record this value in their diary. In case the measurement was markedly different from the blood pressure usually taken at home, they were advised to repeat the measurement a second time to confirm the value. Home pulse pressure (HPP) was calculated by subtracting diastolic BP from systolic BP. For this analysis, monthly average of all HPP values taken at home was considered. HPP variability was computed via the sample standard deviation (SD) of all HPP values. Dialysis adequacy was expressed by weekly Kt/V. Blood sampling was performed at baseline in the early morning after an overnight fast. Cholesterol, hemoglobin, PTH, calcium, phosphate, and C-reactive protein (CRP) were measured using standard methods in the routine clinical laboratory.

### 2.3. Study End-Points

As HPP was measured in a month period, follow-up started the day of the last measurement. All-cause mortality and a combination of mortality and fatal and non-fatal cardiovascular events were the study end-points. Cardiovascular events were classified as follows: stroke (ischemic or hemorrhagic), documented by computed tomography, magnetic resonance imaging, and/or clinical and neurological evaluation; transient ischemic attacks (TIA); myocardial infarction confirmed by serial changes of ECG and cardiac biomarkers; ECG-documented angina episodes; heart failure, diagnosed on the basis of clinical and instrumental evidence, as reported before; ECG documented arrhythmia; peripheral ischemia or amputations; unexpected, sudden death highly suspected as of cardiac origin. Each cause of death was assessed by three independent physicians. In doubtful cases, diagnosis was attributed by consensus. During the review process, involved physician used all available medical information, including hospitalization forms and medical records. In case of death at home, family members and/or general practitioners were interviewed to better understand the circumstances which led to death.

### 2.4. Statistical Analysis

Data were expressed as mean ± standard deviation (normally distributed data), median and inter-quartile range (non-normally distributed data), or as percent frequency (categorical data).

Pearson’s correlation coefficient was used to evaluate the association between HPP and the main demographic, somatometric, and clinical variables (age, sex, cardiovascular events, laboratory data, and therapy). The relationship between blood pressure values (HPP, SBP, DBP, and PP variability) and cardiovascular comorbidities was tested by univariate and multivariate logistic regression analysis, adjusted for traditional and non-traditional confounding factors (age, gender, diabetes, dialysis adequacy, and systolic or diastolic arterial pressure). Univariate and multivariate Cox regression analyses, adjusted for age, gender, diabetes, systolic or diastolic arterial pressure, and dialysis adequacy, were used to assess the association between blood pressure values and mortality or the combined outcome. The effect of age and sex on the association between BP values and mortality/combined outcome was verified, including in the Cox model blood pressure values (HPP, SBP, DBP, BPP variability), age or sex, and their multiplicative term. Statistical analysis was performed via standard statistical packages (SPSS for Windows, Version 26, Chicago, IL, USA; STATA for Windows, Version 16, College Station, TX, USA).

## 3. Results

**Figure 1:**
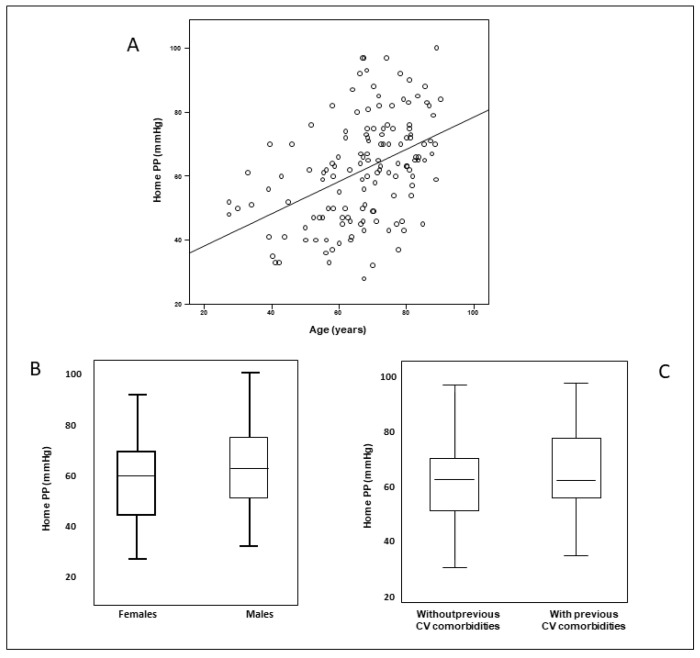
Positive, significant association between HPP and age (**A**), male gender (**B**), and previous cardiovascular events (**C**).

The main clinical, demographic, and somatometric baseline characteristics of the study population are reported in Table 1. HPP was on average 62 ± 16 mmHg, with systolic and diastolic BP of 139 ± 16 mmHg and 77 ± 10 mmHg, respectively. Eighty-three patients were males (59.3%), the mean age was 67 ± 14 years, and BMI was 27 ± 4 kg/m^2^. Overall, 10% of the patients were current smokers. Regarding the most important comorbidities, 36.4% of patients were diabetic, while 47.1% had previous cardiovascular events. The list of the main correlates of HPP is reported in Table 2. Correlation analysis showed a positive, significant association between HPP and age (r = 0.44, *p* < 0.001), male gender (r = 0.21, *p* = 0.01), and previous cardiovascular events (r = 0.28, *p* = 0.001) (Figure 1).

The association between HPP and previous cardiovascular comorbidities was confirmed at univariate logistic regression analysis, where for each 5-unit increase in HPP, a 4% increase in the odds of cardiovascular disease was observed (OR: 1.22, 95% CI: 1.08–1.38, *p* = 0.001). This association was confirmed after adjustment for potential confounders such as age, gender, diabetes, and dialysis adequacy (OR: 1.21, 95% CI: 1.04–1.42, *p* = 0.02). This association was no longer significant after introducing the same model systolic arterial pressure (OR: 1.22, 95% CI: 0.95–1.57, *p* = 0.13).

Differently from HPP, pulse pressure variability was not associated with previous cardiovascular comorbidities, neither at univariate (OR for the 5-unit increase: 1.05, 95% CI: 0.98–1.05, *p* = 0.15) or multivariate analysis (OR for the 5-unit increase: 1.05, 95% CI: 0.98–1.11, *p* = 0.15).

The same analysis performed on the two components of HPP reported a positive, significant association between SBP and cardiovascular comorbidities (univariate: OR for the 5-unit increase: 1.14, 95% CI: 1.02–1.29, *p* = 0.03; multivariate: OR for the 5-unit increase: 1.15, 95% CI: 1.00–1.33, *p* = 0.06; multivariate + DBP: OR for the 5-unit increase: 1.21, 95% CI: 1.03–1.43, *p* = 0.02). Conversely, the inverse relationship between DBP and cardiovascular comorbidities was not significant (univariate: OR for the 5-unit increase: 0.85, 95% CI 0.70–1.02, *p* = 0.08; multivariate: OR for the 5-unit increase: 0.94, 95% CI 0.76–1.17, *p* = 0.60; multivariate + SBP: OR for the 5-unit increase: 0.82, 95%CI: 0.64–1.06, *p* = 0.82).

### 3.1. Survival Analysis

#### 3.1.1. All-Cause Mortality

During a mean follow-up of 35 months, 62 patients died. In a crude COX regression analysis, a five-unit increase in HPP was associated with a 17% increase in the hazard ratio of mortality (HR: 1.17, 95% CI 1.08–1.26, *p* < 0.001) (Table 3). This result was confirmed in a multiple Cox model adjusted for age, gender, diabetes, systolic arterial pressure, and dialysis adequacy (HR: 1.31, 95% CI: 1.12–1.52, *p* = 0.001) (Table 3). Forcing, in the model, previous CV events as potential confounders, the association remained statistically significant (HR: 1.30, 95% CI: 1.11–1.51, *p* = 0.001).

The predictive power of HPP overcomes that of SBP and pulse pressure variability for all-cause mortality. As a result of our analysis, the association between these BP values and the considered outcome was not significant both at univariate (HR for 5-unit increase: 1.04, 95% CI: 0.96–1.14, *p* = 0.33) and multivariate analysis (HR for 5-unit increase: 1.05, 95% CI: 0.94–1.17, *p* = 0.39). Adding previous cardiovascular comorbidities in the model did not change the result (HR for the 5-unit increase: 1.04, 95% CI: 0.93–1.16, *p* = 0.47).

As expected, DBP was protective for all-cause mortality (univariate analysis: HR for the 5-unit increase: 0.70, 95% CI: 0.62–0.80, *p* < 0.001; multivariate analysis: HR for the 5-unit increase: 0.77, 95% CI: 0.66–0.89, *p* = 0.001; multivariate analysis + previous cardiovascular comorbidities: HR for the 5-unit increase: 0.77, 95% CI: 0.66–0.90, *p* = 0.001).

Finally, no association was found between pulse pressure variability and all-cause mortality (univariate analysis: HR for the 5-unit increase: 0.99, 95% CI: 0.97–1.03, *p* = 0.91; multivariate analysis: HR for the 5-unit increase: 0.98, 95% CI: 0.92–1.03, *p* = 0.37; multivariate analysis + previous cardiovascular comorbidities: HR for the 5-unit increase: 0.97, 95% CI: 0.92–1.03, *p* = 0.30).

**Figure 2:**
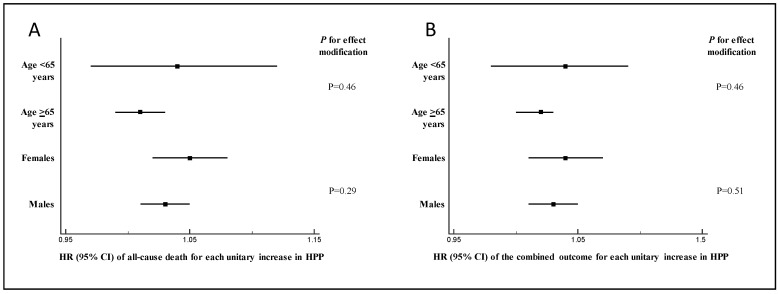
Forest plot showing the absence of effect modification by age and sex on the link HPP-mortality (**A**) and HPP and the combined event (**B**).

No effect modification by age or sex was found on the link between HPP and mortality (*p* for effect modification >0.22) (Figure 2A) or on the association between the other BP measurements (SBP, DBP, and PP variability) and the same outcome (all *p* > 0.05).

#### 3.1.2. Combined Outcome—Death and Cardiovascular Events

During the follow-up of 35 months, 66 patients experienced the combined event of death/CV events. Survival analyses considering the combined event death-CV events as outcome showed similar results. In a crude Cox regression analysis, HPP was directly and strongly related to the combined outcome (HR: 1.17, 95% CI: 1.08–1.26, *p* < 0.001) (Table 4). In strict parallelism with the results obtained with mortality, such an association remained significant also after adjustment for potential confounders such as age, gender, diabetes, systolic arterial pressure, and dialysis adequacy (HR: 1.28, 95% CI: 1.11–1.47, *p* = 0.001) (Table 4). By also adding the previous CV events as potential confounders to the previous adjusted COX model, the association remained statistically significant (HR: 1.25, 95% CI: 1.08–1.44, *p* = 0.003). DBP confirmed the protective role for the combined outcome both at univariate and multivariate analysis (univariate analysis: HR for the 5-unit increase: 0.74, 95% CI: 0.65–0.84, *p* < 0.001; multivariate analysis: HR for the 5-unit increase: 0.78, 95% CI: 0.68–0.91, *p* = 0.001; multivariate analysis + previous cardiovascular comorbidities: HR for the 5-unit increase: 0.78, 95% CI: 0.68–0.91, *p* = 0.001).

No association was found between SBP (univariate analysis: HR for the 5-unit increase: 1.05, 95% CI: 0.97–1.14, *p* = 0.19; multivariate analysis: HR for the 5-unit increase: 1.06, 95% CI: 0.97–1.17, *p* = 0.22; multivariate analysis + previous cardiovascular comorbidities: HR for the 5-unit increase: 1.05, 95% CI: 0.95–1.15, *p* = 0.34) and pulse pressure variability and the combined outcome (univariate analysis: HR for the 5-unit increase: 0.99, 95% CI: 0.97–1.03, *p* = 0.84; multivariate analysis: HR for the 5-unit increase: 0.98, 95% CI: 0.93–1.03, *p* = 0.49; multivariate analysis + previous cardiovascular comorbidities: HR for the 5-unit increase: 0.98, 95% CI: 0.93–1.03, *p* = 0.44).

No effect modification by age or sex was found on the link between HPP and the combined outcome (*p* for effect modification >0.51, Figure 2B) or on the association between the other BP measurements (SBP, DBP, and PP variability) and the same outcome (all *p* > 0.05).

## 4. Discussion

In this study, we found a positive, significant association between HPP and clinical outcomes (all-cause mortality and the combined outcome mortality and fatal/non-fatal CV events), and these associations held true after adjustment for systolic blood pressure and previous cardiovascular comorbidities. HPP was also shown to be a better predictor of clinical outcomes than SBP and pulse pressure variability, whereas DBP confirmed its predictive role for the same outcomes.

Pulse pressure, an index of the pulsatile component of the cardiac cycle [53], has been associated with an increased risk of major cardiovascular events and all-cause and CV mortality in the general population [54,55,56,57,58]. Different from conventional blood pressure measurements, which present a U-shaped or reverse-J relationship with mortality in hemodialysis patients [11,14,27,59], pulse pressure has proven to have a strong and direct association with mortality in these patients [51,52], even after adjustment for systolic blood pressure [51]. In these patients, the capability of PP in predicting cardiovascular events such as ischemic heart disease seems superior to that of systolic pressure [58,60], even though, due to the high correlation between these two measurements, it is difficult to determine their effective contribution [61].

In PD patients, the increase in PP was significantly associated with all-cause mortality [45,47,52,62], cardiovascular mortality [45,52,62], and cardiovascular events [62], even after adjustment for SBP. Nevertheless, a large cohort study involving 2770 patients found an inverse association between PP short-term mortality and a direct one with long-term increased mortality [46]. In addition, in a large Chinese cohort (>7000 PD patients), higher PP (>60 mmHg) was associated with an increased risk of all-cause but not CV mortality [63]. However, all data shown so far refers to standardized pulse pressure measurements, i.e., blood pressure measurements taken at the dialysis unit via a standardized protocol.

In contrast with the other studies, we focused our analysis on BP measurements taken at home, thus without a standardized protocol, within the period of a month.

Unsurprisingly, we found a strong positive association between HPP and age, male gender, and previous cardiovascular events (r from 0.21 to 0.44, *p* < 0.01). The association between HPP and previous cardiovascular comorbidities was independent of a list of potential confounders. This association was no longer significant after introducing the same model of systolic arterial pressure. In survival analysis, a five-unit increase in HPP was associated with a significant 17% (crude analysis) or 31% (adjusted analysis) increase in the hazard ratio of mortality. Similar results were obtained considering the combined death-CV events as an outcome. The predictive power of HPP overcame that of SBP or PP variability, as both failed to predict the considered outcomes. Different from the other BP measurements and in line with the literature, DBP was associated with a reduction of 30% of the risk of mortality and 26% of the risk of death and CV events in this population. Systolic, diastolic, and pulse pressure, though depending on vascular resistance and central artery stiffness, are variously affected by these components. DBP is raised by high peripheral vascular resistance and lowered by high arterial stiffness, so its values and, as a consequence, those of pulse pressure depend on the relative contribution of these factors. In contrast, pulse and systolic pressure are strongly interrelated because both rise with increases in vascular resistance and arterial stiffness [50].

The strong correlation between pulse pressure and arterial stiffness origins from age-related vascular calcification. This process translates into arterial stiffening, which in turn causes larger forward wave amplitude, earlier reflected wave arrival, and greater pulse pressure [64,65]. For this reason, although not a direct measure of arterial stiffness, pulse pressure is usually used as a surrogate marker of arterial compliance [66]. Taken together, this data suggests that the direct associations of HPP with clinical outcomes may be a direct consequence of arterial aging, a well-established cardiovascular risk predictor among people in dialysis patients [67,68].

The same dependence of SBP and PP from vascular resistance and arterial stiffness may explain why, when put together in our models, they remain both significantly associated with all-cause mortality and with the combined event mortality/CV events.

The main limitation of our study is the small sample size. However, with a follow up of about 3 years and almost half of the patients experiencing the event of interest, our cohort can be considered satisfactory. Secondly, enrolled patients were followed up in a single dialysis center, and this prevented our results from being generalized. Third, the overall number of the events collected was relatively small, thus limiting the number of potential confounders to be included in the models. Furthermore, smoking habits were available only in a limited number of subjects. As a further limitation, data about aortic regurgitation was not available in our dataset, so we were not able to exclude from the analysis patients affected by this comorbidity.

In spite of these limitations, our study is the first to show that home blood pressure, measured within a period of a month, is linearly associated with death and cardiovascular events in clinical outcomes and that this association is independent of SBP.

## 5. Conclusions

In conclusion, home BP monitoring is considered a more suitable option for the management of hypertension in dialysis patients, and home pulse pressure represents, in part, arterial stiffness, and it is strongly related to all-cause mortality in peritoneal patients. In these high cardiovascular risk populations, multiple approaches, drug therapy associated with a better volume status control, lifestyle, and sodium and water intake are important to maintain optimal blood pressure control, but it is fundamental to consider all other cardiovascular risk indicators, such as pulse pressure. Home pulse pressure measurement is easy and feasible and can add important information to identify and manage high-risk patients.

## Figures and Tables

**Table 1:** Main demographic, somatometric, and clinical characteristics of the study population.

	Whole Group (*n* = 140)
Age (years)	67 ± 14
BMI (kg/m^2^)	27 ± 4
Male sex *n*. (%)	83 (59.3)
Current smokers *n*. (%)	14 (10)
Past smokers *n*. (%)	35 (25)
Diabetics *n*. (%)	51 (36.4)
With cardiovascular comorbidities ^1^ *n*. (%)	66 (47.1)
On anti-hypertensive treatment *n*. (%)	106 (75.7)
Home Pulse Pressure (mmHg)	62 ± 16
Systolic Blood Pressure (mmHg)	139 ± 16
Diastolic Blood Pressure (mmHg)	77 ± 10
Cholesterol (mg/dL)	171 ± 40
Hemoglobin (g/dL)	11 ± 1.6
PTH (pg/mL)	202 ± 173
CRP (mg/L)	3.7 (3.2–10.3)
Calcium (mg/dL)	8.9 ± 1.3
Phosphate (mg/dL)	5.6 ± 1.6
Kt/V	1.9 ± 0.4

^1^ Cardiovascular comorbidities: The presence, at baseline, of at least one of these comorbidities: angina, myocardial infarction, stroke, TIA, arrhythmia, peripheral arterial disease. Data are expressed as mean ± SD. median and inter-quartile range or as percent frequency, as appropriate.

**Table 2:** Main demographic and clinical correlates of pulse pressure.

Variables	R	*p*
Age (years)	0.435	<0.001
Previous cardiovascular comorbidities	0.234	0.006
Diabetes	0.357	<0.001
NYHA Score	0.281	0.001
Kt/V	−0.147	0.083
Hemoglobin	−0.114	0.182
C-reactive protein	0.053	0.548
Phosphate	−0.162	0.057
PTHi	−0.066	0.449
VitD	0.057	0.501
Use of blood-pressure-lowering therapy	0.257	0.003
Use of erythropoiesis-stimulating agents	−0.105	0.218
Lipid-lowering therapy	−0.053	0.532

**Table 3:** Univariate and multivariate survival analysis showing the association between pulse pressure and all-cause mortality.

Variables (Units of Increase)	Crude Analysis	Fully Adjusted Analysis	Over-Adjusted Analysis
Pulse Pressure (5 mmHg)	1.17 (1.08–1.26), *p* < 0.001	1.31 (1.12–1.52), *p* = 0.001	1.30 (1.11–1.51), *p* = 0.001
Age (1 year)		1.05 (1.01–1.08), *p* = 0.004	1.04 (1.01–1.08), *p* = 0.005
Gender (0 = female; 1 = male)		0.82 (0.44–1.53), *p* = 0.53	0.79 (0.42–1.47), *p* = 0.45
Diabetes (0 = no; 1 = yes)		0.88 (0.51–1.51), *p* = 0.63	0.93 (0.93–1.62), *p* = 0.80
Kt/V (1 unit)		1.01 (0.51–2.01), *p* = 0.97	1.01 (0.51–1.99), *p* = 0.98
Systolic Blood Pressure (1 mmHg)		0.96 (0.93–0.99), *p* = 0.009	0.96 (0.93–0.99), *p* = 0.01
Cardiovascular comorbidities (0 = no; 1 = yes)			1.28 (0.72–2.28), *p* = 0.40

**Table 4:** Univariate and multivariate survival analysis showing the association between pulse pressure and the combined event of death/cardiovascular events.

Variables (Units of Increase)	Crude Analysis	Fully Adjusted Analysis	Over-Adjusted Analysis
Pulse Pressure (1 mmHg)	1.17 (1.08–1.26), *p* < 0.001	1.28 (1.11–1.47), *p* = 0.001	1.25 (1.08–1.44), *p* = 0.003
Age (1 year)		1.04 (1.01–1.06), *p* = 0.01	1.04 (1.01–1.06), *p* = 0.01
Gender (0 = female; 1 = male)		0.98 (0.54–1.74), *p* = 0.93	0.87 (0.48–1.58), *p* = 0.65
Diabetes (0 = no; 1 = yes)		0.93 (0.55–1.56), *p* = 0.77	1.01 (0.60–1.70), *p* = 0.98
Kt/V (1 unit)		0.74 (0.37–1.49), *p* = 0.40	0.67 (0.33–1.35), *p* = 0.26
Systolic Blood Pressure (1 mmHg)		0.96 (0.94–0.99); *p* = 0.01	0.97 (0.94–0.99); *p* = 0.02
Cardiovascular comorbidities (0 = no; 1 = yes)			1.60 (0.92–2.81), *p* = 0.10

## Data Availability

The data presented in this study are available on request from the corresponding author. The data are not publicly available as related to sensitive data for which informed consent was needed.
